# Dietary Polyphenols Protect Against Oleic Acid-Induced Steatosis in an in Vitro Model of NAFLD by Modulating Lipid Metabolism and Improving Mitochondrial Function

**DOI:** 10.3390/nu11030541

**Published:** 2019-03-03

**Authors:** Hossein Rafiei, Kosar Omidian, Brian Bandy

**Affiliations:** Nutrition Division, College of Pharmacy and Nutrition, University of Saskatchewan, Saskatoon, SK S7N 2Z4, Canada; hossein.rafiei@usask.ca (H.R.); kosar.omidian@usask.ca (K.O.)

**Keywords:** mitochondrial dysfunction, mitochondrial membrane potential, NAFLD, polyphenols, steatosis, bioenergetics

## Abstract

In this study, we aimed to determine the relative effectiveness of common dietary polyphenols or the isoquinoline alkaloid berberine in protecting against molecular mechanisms underlying non-alcoholic fatty liver disease (NAFLD) involving changes to cellular lipid metabolism and bioenergetics. In a model of steatosis using HepG2 hepatocytes, exposure of the cells to 1.5 mM oleic acid (OA) for 24 h caused steatosis and distorted cell morphology, induced the expression of mRNA for enzymes that are involved in lipogenesis and fatty acid oxidation (*FAS* and *CPT1A*), and impaired indices of aerobic energy metabolism (*PPARγ* mRNA expression, mitochondrial membrane potential (MMP), and galactose-supported ATP production). Co-treatment with 10 µM of selected polyphenols all strongly protected against the steatosis and changes in cell morphology. All polyphenols, except cyanidin, inhibited the effects on *FAS* and *PPARγ* and further increased *CPT1A1* expression, suggesting a shift toward increased β-oxidation. Resveratrol, quercetin, catechin, and cyanidin, however not kuromanin or berberine, ameliorated the decreases in MMP and galactose-derived ATP. Berberine was unique in worsening the decrease in galactose-derived ATP. In further investigations of the mechanisms involved, resveratrol, catechin, and berberine increased SIRT1 enzyme activity and p-AMPKα^Thr172^ protein, which are involved in mitochondrial biogenesis. In conclusion, selected polyphenols all protected against steatosis with similar effectiveness, however through different mechanisms that increased aerobic lipid metabolism and mitochondrial function.

## 1. Introduction

Non-alcoholic fatty liver disease (NAFLD) occurs following the ectopic accumulation of fat in the liver which is the manifestation of an imbalance between lipid influx and removal mechanisms [1]. Initially a benign condition, NAFLD can progress to non-alcoholic steatohepatitis (NASH), fibrosis, and cirrhosis [2]. The “two-hit” hypothesis suggests steatosis as the “first hit”, making the liver susceptible to “second hits” including oxidative stress, gut-derived endotoxins, or pro-inflammatory cytokines [3]. Oleic and palmitic acid are the most abundant fatty acids in the hepatic triglycerides of healthy subjects and patients with NAFLD and contribute to the pathogenesis of the disease [4]. Oleic acid has been shown to be more steatotic than palmitic acid [5] and to induce reactive oxygen species (ROS) generation and *TNFα* expression [6] which both contribute to the progression of NAFLD to NASH. 

Hepatic lipid metabolism is delicately regulated by the interaction among different transcription factors, including peroxisome proliferator-activated receptors (PPARs) and nuclear receptors, and genes involved in lipogenesis and fatty acid β-oxidation [7]. Any imbalances in these intertwined and precisely regulated pathways may promote NAFLD/NASH. Although mitochondrial fatty acid β-oxidation increases in diet-induced obesity and insulin resistance [8,9], it cannot proportionately metabolize excess fat and its capacity may be disrupted by mitochondrial dysfunction and oxidative phosphorylation (OXPHOS) incompetence [10].

Recently, mitochondrial dysfunction has been implicated in the progression of simple steatosis to NASH [11,12]. Mitochondrial dysfunction has a key role in the progression of NAFLD. Due to the pivotal role of mitochondria in lipid metabolism, ROS and ATP production, and apoptosis [13], mitochondrial dysfunction is suggested to contribute to the severity of NAFLD/NASH [14]. The mitochondrial dysfunction also lowers the mitochondrial membrane potential (MMP) [15], further exacerbating NAFLD. Due to impaired oxidative phosphorylation, hepatic ATP stores, which are critical in maintaining the integrity of hepatic tissue, become depleted in NAFLD and NASH, further worsening the condition [16]. Higher ROS production and consequent mitochondrial DNA damage further decreases mitochondrial biogenesis in NAFLD, producing fewer functional mitochondria in hepatocytes (reviewed in [17]). Therapies to induce mitochondrial biogenesis may therefore facilitate metabolizing excess fat and, thus, may alleviate NAFLD/NASH [18].

One class of compounds that may provide therapeutic effects against NAFLD and progression to NASH is dietary polyphenols. Found in our diet in foods such as fruits and vegetables and beverages such as teas, hot chocolate, coffee, and red wine [19], polyphenol-rich foods, beverages, or extracts have previously been observed to protect against features of NAFLD in animal models [20,21] and humans [21]. However, it is not known which polyphenols are the most effective in the prevention of NAFLD and by what molecular mechanisms they act.

Different polyphenols may protect by different mechanisms. The flavonoid classes of polyphenols, such as anthocyanins, flavonols (eg. quercetin), and flavanols (catechins), are well known as strong antioxidants [22]. We previously showed that in hepatocytes exposed to OA (oleic acid), different polyphenols all inhibited an increase in intracellular ROS and most prevented an increase in the expression of the pro-inflammatory cytokine TNFα [6]. The polyphenols resveratrol and quercetin and the O-methylated phenol berberine were also able to ameliorate an OA-induced decrease in cellular mitochondrial content. 

Part of the mechanism by which polyphenols act may be by increasing aerobic lipid metabolism and mitochondrial bioenergetics. In animal studies, for example, a polyphenol-rich grape skin extract or sweet cherry anthocyanins decreased liver intracellular lipid accumulation by improving hepatic lipid metabolism [23,24]. In other animal studies on NAFLD, polyphenol-rich foods/extracts such as purified anthocyanins from bilberry and black currant [25], green tea extract [26], and virgin olive oil [27], or supplementation with resveratrol [28] or quercetin [29], have been shown to improve mitochondrial function. However, the relative effectiveness of different polyphenols and the mechanisms behind the effects on lipid metabolism and mitochondrial function are not known.

The aim of the present study was to compare different classes of polyphenols for their abilities to preserve HepG2 cell morphology, prevent OA-induced lipid accumulation and mitochondrial dysfunction, and induce mitochondrial biogenesis and bioenergetics. 

## 2. Materials and Methods

### 2.1. HepG2 Cell Culture Conditions

Prior to the experiments, HepG2 cells were grown in multi-well plates in DMEM (5.5 mM glucose and 2 mM glutamine) with 10% fetal bovine serum (FBS) and 1% penicillin/streptomycin for 24 h at 37 °C, 5% CO_2_. In some experiments on intracellular ATP levels, the cells were grown in DMEM with 25 mM galactose instead of glucose.

### 2.2. Treatment with OA and Polyphenols

HepG2 cells were treated with OA (Sigma-Aldrich, Oakville, ON, Canada) and different polyphenols, as previously described [6]. Briefly, after growing HepG2 cells in 96-well plates for 24 h, the cells were treated with 10 µM polyphenol (resveratrol, quercetin, catechin, berberine (Sigma-Aldrich, Oakville, ON, Canada), cyanidin, or cyanidin-3-glucoside (kuromanin) (Extrasynthese, Genay Cedex, France)) for 2 h followed by 1.5 mM OA (dissolved in medium with 1% bovine serum albumin) for 24 h. 

### 2.3. Measuring Intracellular Lipid Content

Intracellular lipid content was determined using Nile Red dye (MP Biomedical, Santa Ana, CA, USA) as previously described [30]. After treatments for 24 h, the medium was replaced with medium containing Nile Red at 1 µg/mL and the cells were incubated in the dark for 30 min at 37 °C. The cells were then carefully washed two to three with phosphate buffered saline (PBS) and fluorescence was determined in a plate reader at 488 nm excitation and 585 nm emission. Fluorescence images were captured using a ZOE Fluorescence Imager (Bio-Rad, Mississauga, ON, Canada).

### 2.4. RT-qPCR

The expression of the genes in HepG2 cells was determined using RT-qPCR as previously described [6]. Briefly, following the treatment of HepG2 cells with polyphenols for 2 h and OA for 24 h, total RNA was extracted using TRIzol reagent and RNAeasy Mini Kit (Qiagen, Toronto, ON, Canada) according to the manufacturer’s instructions. Reverse transcription of 2 μg of total RNA to complementary DNA (cDNA) was performed in 20 µL reactions using a VILO cDNA synthesis kit (Invitrogen, USA) and a Thermocycler (Bio-Rad, Mississauga, ON, Canada). All primers were synthesized by Integrated DNA Technologies Company (IDT, Canada) (Supplemental Table S1). The PCR was performed using a PCR system (ABI 7300; Applied Biosystems, Mississauga, ON, Canada) using power SYBR green real-time PCR master mix (Applied Biosystems, Mississauga, ON, Canada), according to the manufacturer’s protocol. Relative mRNA expression was determined by a comparative method (2^−ΔΔCT^) using GAPDH as a reference gene. All results were normalized to GAPDH.

### 2.5. Quantification of Intracellular ATP

ATP content was quantified using the CellTiter-Glo Luminescent Cell Viability Assay kit (Promega, Madison, WI, USA) based on the manufacturer’s instructions. Briefly, 1 × 10^4^ cells per well were grown in white 96-well clear-bottom plates with either glucose or galactose for 24 h and the cells were then treated with polyphenols for 2 h and OA for 24 h or 72 h. The cells were lysed using the reagent included in the kit, and after vigorous shaking and incubating at room temperature for 10 min, the luminescence of each well was read using a microplate reader.

### 2.6. SIRT1 Deacetylase Activity

SIRT1 activity was measured using a SIRT1 direct fluorescence screening assay kit (Cayman Chemical, Ann Arbor, MI, USA). The assay was performed by incubation of 5 µL of protein lysates with 15 µL substrate solution containing fluorogenic acetylated p53 peptide sequence (Arg-His-Lys-Lys(ε-acetyl)-AMC) and co-substrate NAD+, and 25 µL assay buffer. The plate was covered and incubated on a shaker at room temperature for 45 min and then 50 µL of developer was added to each well and the plate was incubated at room temperature for another 30 min to release AMC from the deacetylated peptide. Using a fluorescence reader, the intensity of fluorescence was measured at excitation 360 nm and emission detection at 460 nm. Protein concentration in samples was quantified using a bicinchoninic acid (BCA) protein assay kit and the results of SIRT1 activity were normalized to protein concentrations.

### 2.7. Western Blotting

After growing HepG2 cells and following treatment with polyphenols and OA, total protein was extracted using ice-cold RIPA buffer (Millipore, Etobicoke, ON, Canada) supplemented with Halt protease and phosphatase inhibitor and EDTA (Thermo Fisher, Ottawa, ON, Canada). After centrifugation at 14,000 rpm for 15 min, supernatant was obtained and protein content was quantified using BCA protein assay. Protein samples were then normalized, mixed with loading buffer, and denatured by heating at 70 °C for 10 min. Then, 40 micrograms of protein was loaded into each well of Novex Wedge Well Pre-Cast gels (Invitrogen, Burlington, ON, Canada) and was then transferred to a 0.45 µm nitrocellulose membrane. The membrane was blocked with Tris-buffered saline containing Tween-20 (TBST) and 5% bovine serum albumin (BSA) for 1 h at room temperature. The membrane was then incubated overnight at 4 °C with rabbit primary monoclonal antibodies for phospho-AMPKαThr172 (1:500) and β-actin (1:500) (both from Cell Signaling Technology, Danvers, MA, USA), and PGC-1α (1:500, Abcam, Toronto, ON, Canada). Membranes were then washed with Tris-buffered saline with 0.1% Tween-20 and incubated for 1h with secondary anti-rabbit antibody (1:2000, Santa Cruz Biotechnology, Santa Cruz, CA, USA). After several washings, the membrane was incubated with the enhanced chemiluminescence western blotting detection kit (Thermo Fisher, Ottawa, ON, Canada) and the bands were quantified using an Alpha Imager Gel Imaging System (Alpha Innotech, San Leandro, CA, USA). The results were normalized to their corresponding control β-actin.

### 2.8. Mitochondrial Membrane Potential (MMP)

MMP was measured using tetramethlyrhodamine ethyl ester (TMRE) (Invitrogen, Burlington, ON, Canada), as previously described [30]. After growing HepG2 cells at 7.5 × 10^4^ per well, the cells were treated with 10 µM polyphenols for 2 h and 1.5 mM OA for 24 h. The cells were then washed carefully one to two times with Hanks’ buffer and incubated with 500 nM TMRE for 30 min at 37 °C in the dark. Excess dye was then removed by washing with Hanks’ buffer and fluorescence was measured with a Synergy HT plate reader at an excitation wavelength of 549 nm and an emission wavelength of 575 nm. The results are reported as fluorescence intensity and are compared to the relative controls. The fluorescence images of MMP were captured using a ZOE Fluorescence Imager (Bio-Rad, Mississauga, ON, Canada).

### 2.9. Statistical Analysis

Results were expressed as mean values with their standard error of means (SEM) for the number of experiments indicated in every case. The difference between groups was first analyzed with one-way ANOVA and Dunnett’s post hoc test was used to compare the mean of each group with the OA alone condition. The level of significance was *p* < 0.05.

## 3. Results

### 3.1. HepG2 Cell Morphology

**Figure 1:**
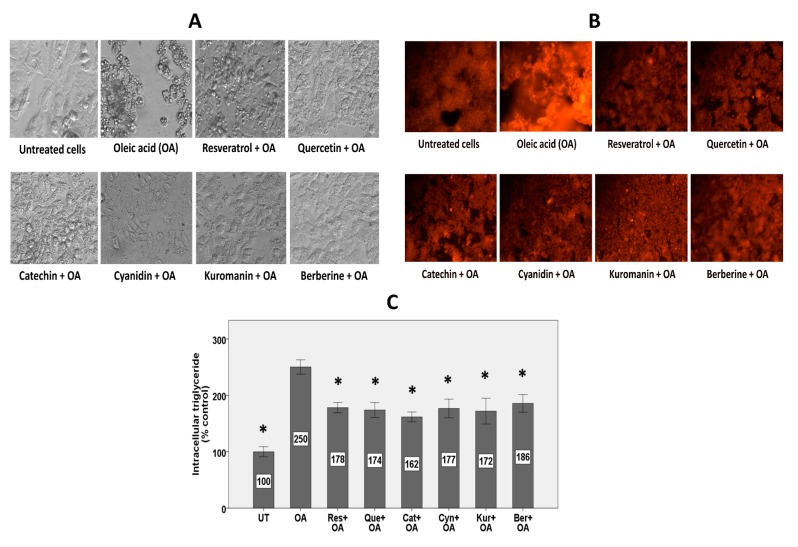
The effect of polyphenols on OA-induced steatosis. HepG2 cells were treated with 10 µM polyphenols for 2 h followed by treatment with 1.5 mM OA for 24 h in the presence of the polyphenols. (**A**) Brightfield images with a 40× objective lens showing morphology of hepatocytes (**B**) Fluorescence images with a 40× objective lens using Nile Red fluorescence probe showing intracellular lipid content. (**C**) Intracellular lipid after quantification of Nile Red fluorescence using a microplate reader. Lipid levels were normalized to protein level in each treatment group as determined by the bicinchoninic acid (BCA) protein assay. Untreated cells were set at 100% and data are presented as % of the untreated cells. The Figure represents means ± SEM of three independent experiments with three wells of cells in each experiment. UT: untreated, OA: oleic acid, Res: resveratrol, Que: quercetin, Cat: catechin, Cyn: cyanidin, Kur: kuromanin, Ber: berberine. The data were analyzed by one-way ANOVA with Dunnett’s post-hoc comparison to the OA alone condition. (*) Significantly different from the OA alone condition at *p* < 0.05.

While untreated HepG2 cells showed a healthy eye-shaped morphology, cells treated with OA showed morphological distortion with numerous droplets of fat (Figure 1A). Co-treatment with all polyphenols could protect against OA-induced lipid accumulation and consequent morphological distortion (Figure 1A). Fat droplets in the HepG2 cells treated with polyphenols looked smaller and more dispersed and the cells preserved their eye-shaped morphology compared to the OA alone condition in which the cells were rounded, swollen, or distorted in morphology.

### 3.2. Intracellular Lipid Accumulation

Preliminary dose-response studies with the selected polyphenols at physiologically relevant concentrations (1, 5, or 10 µM) showed strong protection against steatosis and reactive oxygen species (ROS) generation by the highest concentration and because of this, we conducted the study with a concentration of 10 µM. As shown in Figure 1B, HepG2 cells exposed to OA showed higher intracellular fat compared to untreated cells. Different polyphenols reduced the fluorescence intensity, representing inhibition of OA-induced lipid accumulation. These results are in accordance with the results shown in Figure 1A. Quantification of the results using a microplate reader showed 150% higher lipid content in HepG2 cells exposed to OA, while co-treatment with 10 µM polyphenols inhibited accumulation of lipids by 42–58% (Figure 1C).

### 3.3. Mitochondrial Membrane Potential

**Figure 2:**
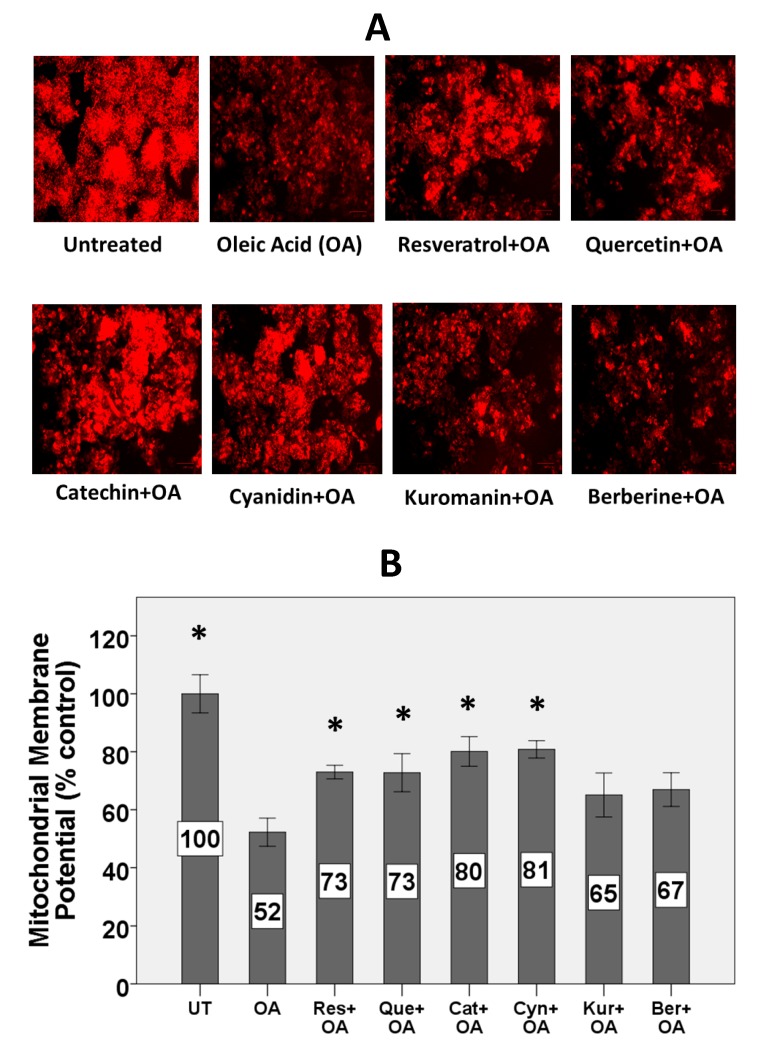
Effect of OA and polyphenols on mitochondrial membrane potential. HepG2 cells were treated with 10 µM polyphenols for 2 h followed by 1.5 mM OA for 24 h. (**A**) Fluorescence images representing the effect of OA and polyphenols on MMP after TMRE staining. Images at 20x magnification were captured using a ZOE Fluorescent Cell Imager. (**B**) MMP was measured following quantification of TMRE fluorescence using a microplate reader. Untreated cells were set at 100% and data are presented as % of the untreated cells. The Figure represents means ± SEM of three independent experiments with three wells of cells in each experiment. (*) Significantly different from the OA alone condition at *p* < 0.05.

Treatment with OA for 24 h decreased the MMP in HepG2 cells (Figure 2A). Treatment with catechin, resveratrol, quercetin, and cyanidin protected against OA-mediated decline of MMP. 

Quantification of TMRE fluorescence showed that OA significantly decreased the MMP by 48% (Figure 2B). Treatment with the polyphenols cyanidin, catechin, quercetin, and resveratrol, however not berberine and kuromanin, protected by 44–60% against the OA-induced decrease of MMP.

### 3.4. Expression of Genes Involved in Fatty Acid Β-Oxidation

**Figure 3:**
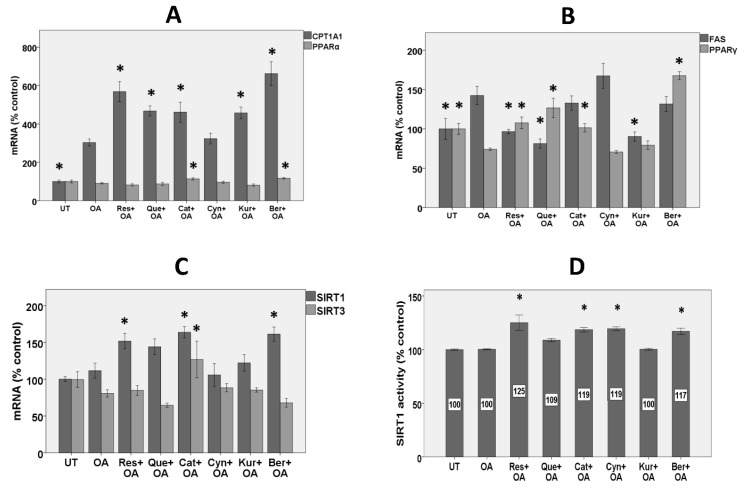
Effects on the genes controlling lipid metabolism and mitochondrial biogenesis, and SIRT1 activity. HepG2 cells were treated with 10 µM polyphenols for 2 h followed by 1.5 mM OA for 24 h in the presence of the polyphenols and mRNA expression for different genes as well as SIRT1 activity were measured. (**A**) Comparison of the effects of polyphenols on the expression of genes involved in fatty acid oxidation. (**B**) Comparison of the effects of polyphenols on lipogenic genes. (**C**) Comparison of the effects of polyphenols on genes involved in mitochondrial biogenesis (**D**) Comparison of the effects of polyphenols on SIRT1 activity. UT: untreated, Res: resveratrol, Que: quercetin, Cat: catechin, Cyn: cyanidin, Kur: kuromanin, Ber: berberine. For SIRT1 activity, data are normalized to protein concentration. Untreated cells were set at 100% and data are presented as % of the untreated cells. The Figure represents means ± SEM of two independent experiments with three wells of cells in each experiment. (*) Significantly different (*p* < 0.05) from the OA alone condition.

OA significantly induced mRNA for carnitine palmitoyltransferase 1 (*CPT1A1*) by 200%, apparently as an adaptive mechanism (Figure 3A). Treatment with berberine, resveratrol, quercetin, catechin, and kuromanin, however not cyanidin, further significantly increased *CPT1A1* expression by 118, 87, 54, 52, and 50%, respectively.

OA did not change *PPARα* mRNA, while only treatment with berberine and catechin, however no other polyphenols, significantly increased *PPARα* expression by 29 and 25%, respectively (Figure 3A).

### 3.5. Expression of Lipogenic Genes

OA significantly increased fatty acid synthase mRNA (*FAS)* in HepG2 cells by 43% (Figure 3B). Resveratrol, quercetin, and kuromanin completely prevented the increase in *FAS* expression. Conversely, OA significantly inhibited *PPARγ* expression by 26%, while berberine, quercetin, resveratrol, and catechin (however not anthocyanins) significantly prevented the decrease by more than 100% (Figure 3B).

### 3.6. Expression of Sirtuins

OA did not change sirtuin1 mRNA (*SIRT1*) expression, while catechin, berberine, and resveratrol (however not quercetin and anthocyanins) significantly induced *SIRT1* mRNA by 46%, 44%, and 35%, respectively (Figure 3C).

Treatment with OA alone did not change the expression of *SIRT3* mRNA, while only catechin increased its expression by 57% compared to the OA alone condition (Figure 3C).

### 3.7. SIRT1 Activity

As with *SIRT1* mRNA expression, OA had no effect on SIRT1 activity, while resveratrol, catechin, and berberine significantly increased SIRT1 activity by 25, 19, and 17%, respectively (Figure 3D). Consistent with no effect on mRNA expression, quercetin and kuromanin had no effect on SIRT1 activity. An exception for a different effect on mRNA expression and SIRT1 activity was cyanidin, which though it had no effect on SIRT1 expression, increased SIRT1 activity by 19%.

### 3.8. Expression of Proteins Involved in Mitochondrial Metabolism and Biogenesis

**Figure 4:**
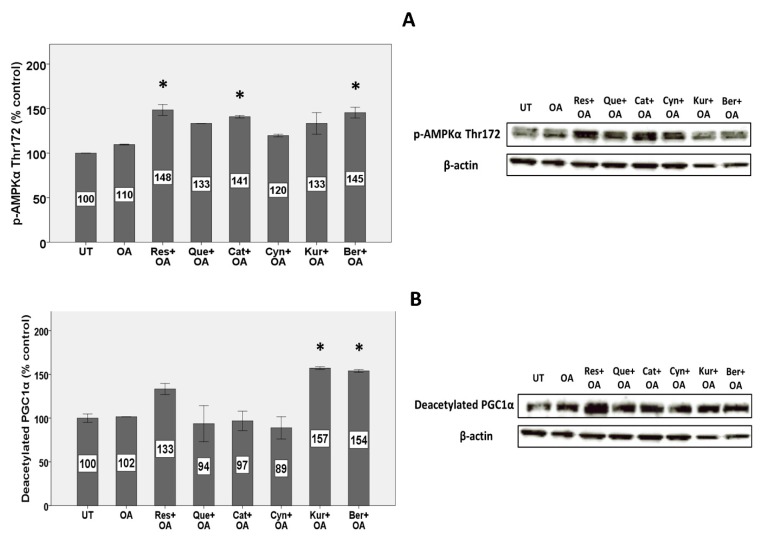
Effects on protein expression of (**A**) p-AMPKα^Thr172^ and (**B**) deacetylated PGC1α. HepG2 cells were treated with 10 µM polyphenols for 2 h and 1.5 mM OA for 24 h, and protein levels were measured by Western blot (*n* = 3). Untreated cells were set at 100% and data are presented as % of the untreated cells. β-actin was used as an internal control. (*) Significantly different (*p* < 0.05) from the OA alone condition.

Treatment with OA did not change the phosphorylation of AMPKα on threonine 172 (p-AMPKα^Thr172^), while some polyphenols such as resveratrol, berberine, and catechin induced the phosphorylation by 35, 32, and 28%, respectively (Figure 4A).

In measurements of deacetylated PGC1α, OA had no effect, while only kuromanin and berberine increased deacetylated PGC1α by 54 and 51%, respectively (Figure 4B).

### 3.9. Intracellular ATP

**Figure 5:**
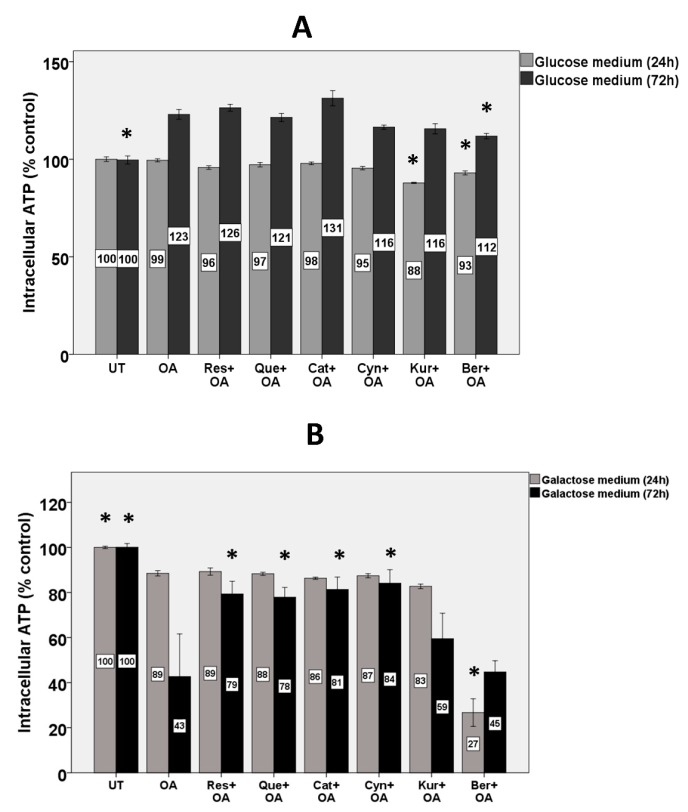
Effects on ATP stores in HepG2 cells grown in glucose and galactose medium. HepG2 cells were grown in (**A**) glucose (5.5 mM) and (**B**) galactose medium (25 mM) and were treated with 10 µM polyphenols for 2 h and 1.5 mM OA for 24 and 72 h. Untreated cells were set at 100% and data are presented as % of the untreated cells. The bars represent means ± SEM of three independent experiments with three wells of cells in each experiment. (*) Significantly different from the OA alone condition at *p* < 0.05.

Intracellular ATP stores in HepG2 cells were quantified after growing the cells in either glucose or, in order to force reliance on mitochondrial energy metabolism, galactose medium. When HepG2 cells were grown in glucose medium for 24 h, OA and polyphenols did not affect ATP stores except for slight (12 and 7%) decreases by kuromanin and berberine, respectively (Figure 5A). In the glucose medium, after 72 h, OA increased ATP stores by 23% and treatment with polyphenols had no effect on ATP stores except a slight (9%) decrease by berberine (Figure 5A).

When the cells were grown in galactose medium for 24 h, OA slightly decreased intracellular ATP stores (by 11%) and polyphenols showed no protection (Figure 5B). Berberine worsened the effect of OA and together decreased ATP stores by 73% compared to untreated cells. In galactose medium, after 72 h, OA depleted intracellular ATP by 57%, while cyanidin, catechin, resveratrol, and quercetin inhibited the decrease by 95, 88, 84, and 81%, respectively (Figure 5B). Berberine and kuromanin did not protect against it.

## 4. Discussion

This study was designed to investigate and compare the effects of different classes of polyphenols on steatosis, lipid metabolism, and mitochondrial bioenergetics in an in vitro model of steatosis. Unlike many in vitro studies that have used high doses of polyphenols, which is not physiologically relevant to concentrations found in blood plasma (≤10 µM), a relatively low dose of polyphenols (10 µM) was selected (according to findings of the preliminary dose-response experiment) and used in this study. The concentrations in portal vein plasma would be higher than in systemic plasma, and this dose is similar to the concentration of quercetin found in portal plasma (16 µM) after intestinal instillation of a quercetin glycoside [31]. The polyphenols chosen were representative of different classes including (1) flavonoids—a flavonol (quercetin), a flavanol (catechin), and anthocyanins (cyanidin and kuromanin), (2) a stilbenoid (resveratrol), and (3) a methoxyphenyl alkaloid (berberine). Although berberine is not itself a polyphenol, it can be demethylated by the activity of cytochrome P450 in hepatocytes to produce an ortho-dihydroxy polyphenolic compound.

### 4.1. Polyphenols All Similarly Protected against Steatosis

In measurements of steatosis, OA increased intracellular lipid accumulation and the different polyphenols all gave similar protection. This is consistent with previous studies showing individual polyphenols do inhibit lipid accumulation in HepG2 cells treated with OA [32,33], palmitic acid [34], or both [35] and shows that different polyphenols are similarly protective.

### 4.2. Polyphenols except Cyanidin Regulated the Expression of Genes and Proteins Involved in Lipid Metabolism

The anti-steatotic effect of polyphenols in the current study may be partly explained by the increased expression of enzymes involved in fat oxidation, such as the hepatic mitochondrial enzyme *CPT1A1*, and decreased expression of lipogenic enzymes, such as *FAS*. However, this mechanism may not be the case for cyanidin, which had no effect on these genes. We previously found cyanidin to increase mRNA for uncoupling protein 2 (*UCP2*) [6], which could partially explain its anti-steatotic effect in this study. 

In the expression of *CPT1A1*, OA and polyphenols acted in the same direction to strongly increase the expression. Increased OA-induced expression of *CPT1A1* in HepG2 cells could be interpreted as an adaptive mechanism by these cells to metabolize excess fat. Consistent with our results, other studies showed that hepatic fatty acid oxidation and CPT1 expression or activity is often enhanced in rodents and patients with NAFLD [11]. The current results showed that all polyphenols except cyanidin further upregulated *CPT1A1* mRNA. These results are consistent with a few previous studies where individual flavonoids or flavonoid-rich extracts were shown to increase mRNA for CPT1 dose-dependently in HepG2 cells treated with free fatty acids [36] or CPT1 expression or activity in genetically obese or high-fat fed rats [37,38]. The strong induction of *CYP1A1* by polyphenols in our study may help explain their anti-steatotic effects through increased mitochondrial β-oxidation of fatty acids.

To gain further insights into the effect on lipid metabolism by polyphenols, we measured the effects on the expression of PPARα. The transcriptional control of genes by PPARα has critical roles in lipid metabolism, inflammation, and fibrosis [39]. Once activated, PPARα induces fatty acid oxidation-related genes, such as CPT1A1 [39]. While many studies have reported upregulated *PPARα* mRNA in NAFLD, others have similarly observed unchanged or even down-regulated *PPARα* in NAFLD [11,40]. Polyphenols also had little effect on PPARα expression in the current study, which is in contrast to some studies in rodents fed polyphenols with a high-fat diet [41]. Some polyphenols such as cyanidin may be an agonist and a natural ligand for different PPARs [42] and, therefore, may affect PPAR activity without affecting PPARα mRNA or protein expression.

Comparison of polyphenols was further investigated by the effects on lipogenic *FAS* and *PPARγ* mRNA expression. OA induced *FAS* by 43%. FAS is an important enzyme in lipogenesis which catalyzes the production of palmitic acid from malonyl-CoA [43]. Consistently, other studies have found increased *FAS* in the liver of high fat-fed mice [44]. Higher *FAS* has also been shown in NAFLD patients [45], and FAS was suggested to be an intriguing therapeutic target for NAFLD [43]. We observed that several polyphenols prevented the increase in the expression of *FAS*. Polyphenols have previously been reported to inhibit steatosis by decreasing mRNA and protein expression of FAS in HepG2 cells treated with oleic [46] or palmitic acid [36]. Although cyanidin decreased intracellular triglyceride in our study, it was unique in having no effect on both *CPT1A1* and *FAS*, which suggests that it may act through other mechanisms.

OA significantly decreased PPARγ expression, while several polyphenols, except anthocyanins, reversed this inhibition. PPARγ induces genes involved in lipid uptake and adipogenesis [47]. While most evidence shows that PPARγ expression increases in diabetic or obesity models of NAFLD [48] and suggest a deleterious and lipogenic role for hepatic PPARγ in NAFLD [49], others suggest a beneficial role for PPARγ [50,51]. While some studies show that treatment of rodents on an obesogenic high-fat diet with polyphenols decreases PPARγ expression in NAFLD [40,52], others are consistent with our study and showed that higher hepatic *PPARγ* induced by polyphenols may be a beneficial outcome in NAFLD, causing improved hepatic insulin signaling and decreased hepatic steatosis [53].

### 4.3. Polyphenols Differently Affected the Expression of Genes and Proteins involved in Mitochondrial Biogenesis

Other aspects of NAFLD and lipid metabolism that we investigated in the current study are those of mitochondrial biogenesis and energy metabolism. Hepatic mitochondrial biogenesis and function are adversely affected in NAFLD, causing inefficient lipid metabolism by hepatocytes [17]. Increasing mitochondrial biogenesis can therefore be a promising approach in the prevention or treatment of NAFLD/NASH. Our results showed that the expression of SIRT1 and SIRT3 and the activity of SIRT1 were not changed following treatment of HepG2 cells with OA. In contrast to our findings, lower protein expression of SIRT1 was reported in rodents with NAFLD [54]. Treatment with resveratrol, catechin, and berberine, however, increased both the gene expression and activity of SIRT1. Different polyphenols such as berberine [55] and resveratrol [56] have previously been shown to induce SIRT1 expression in different models of NAFLD and insulin resistance. SIRT1 induces mitochondrial biogenesis through the deacetylation of PGC1α and activation of AMPK and therefore increases fatty acid β-oxidation that may help to alleviate NAFLD [57]. SIRT1 also inhibits the transcription of lipogenic genes [58]. SIRT1-knockout mice showed lower fatty acid oxidation and higher hepatic steatosis [59]. Among polyphenols, resveratrol has been widely studied with regard to SIRT1 since resveratrol is a potent activator of SIRT1 [60]. In agreement with this property, among all the polyphenols of interest in our study, resveratrol showed the strongest effect on SIRT1 activity.

Consistent with the results on SIRT1 expression and activity, we found higher phosphorylation of threonine 172 on AMPK catalytic α subunit by the same polyphenols (resveratrol, catechin, and berberine). This is in agreement with other studies, showing that flavonoids such as liquiritigenin, an active flavonoid in licorice, could increase phosphorylated AMPK in HepG2 cells and thereby inhibit mitochondrial dysfunction [61]. AMPK activation induces mitochondriogenesis in hepatocytes and leads to increased ATP-producing pathways such as β-oxidation and glycolysis, however it reduces energy-consuming pathways such as lipogenesis [62]. Interestingly, we previously have shown that the same polyphenols (resveratrol, catechin, and berberine) and quercetin induced several genes that are involved in mitochondrial biogenesis [6].

With measurements of deacetylated PGC1α protein, OA had no effect and only kuromanin and berberine produced an increase. PGC1α is a key protein downstream of AMPK and SIRT1 which is involved in mitochondrial biogenesis and is a promising factor in the prevention of NAFLD [57]. PGC1α protein can be activated by either phosphorylation by kinases (such as AMPK) or deacetylation by SIRT1. Since some polyphenols such as catechin had no effect on deacetylation of PGC1α yet increased AMPK phosphorylation in our study, it may be that such polyphenols activate PGC1α by phosphorylation but not deacetylation.

### 4.4. Polyphenols except Kuromanin and Berberine Preserved Mitochondrial Bioenergetics

OA depolarized MMP in HepG2 cells by approximately 50%. Recent investigations show decreased MMP in NASH compared to fatty liver and it was suggested that a lower activity of the mitochondrial respiratory chain could play a major role in this decrease [11]. Depolarization of mitochondria plays a critical role in the initiation of apoptosis and cell death in NAFLD [63]. We showed that polyphenols differ in preserving MMP. Resveratrol, catechin, quercetin, and cyanidin, however not kuromanin and berberine, protected against OA-induced decrease of MMP. Consistent with our results, others have shown that different polyphenols such as curcumin [64] or pomegranate punicalagins [15] can protect against free fatty acid-induced dissipation of MMP. This protection was also shown by flavonoids from *Rosa laevigata Michx* fruit in rodents fed a high-fat diet [65]. Accordingly, preserving MMP may be one of the mechanisms by which polyphenols prevent the progression of simple steatosis to NASH since it is one of the hallmarks of cell survival and functionality of mitochondria.

To further compare the effectiveness of different classes of polyphenols for protection against mitochondrial dysfunction, intracellular ATP stores were measured. Impaired mitochondrial function causes depletion of hepatic ATP stores in NASH [66,67]. The cellular ATP stores are critical in maintaining the integrity of hepatic tissue and preventing hepatocellular injury [68]. Although mitochondrial function in our study was affected by OA due to dissipated MMP, we could not observe any depletion of ATP stores in HepG2 cells grown in glucose medium for 24 and 72 h. Many studies have shown that when mitochondrial oxidative phosphorylation is impaired in cells (particularly cancer cells), they rely on glycolysis to compensate for their reduced mitochondrial-mediated ATP production [69].

Replacing glucose with galactose is a way to shift metabolic pathways toward oxidative phosphorylation which increases the sensitivity and responsiveness to mitochondrial dysfunction [69,70]. Therefore, in order to bypass glycolysis and more appropriately elucidate the effect of mitochondrial dysfunction on ATP production, HepG2 cells were grown in galactose medium. In this medium, treatment with OA for 72 h depleted ATP stores by more than 50%, while treatment with some polyphenols maintained ATP stores. These results are consistent with other studies, showing that treatment of hepatocytes with free fatty acids depletes, while polyphenols preserve ATP stores [64,71]. Interestingly, the results on ATP are consistent with the results of MMP in which resveratrol, quercetin, catechin, and cyanidin, however not kuromanin and berberine, preserved both MMP and ATP stores. Also, in both experiments, OA showed approximately 50% inhibition. It appears that when grown on galactose, ATP stores are dependent on MMP and by inhibiting OA-induced dissipation of MMP and mitochondrial dysfunction, the polyphenols allowed mitochondria to maintain intracellular ATP stores.

A noteworthy finding of the current study was decreased ATP stores induced by berberine in both glucose and especially galactose medium after 24 h. Also, while most polyphenols protected against the OA-induced decline in the mitochondrial membrane potential, berberine did not. A previous study [72] showed berberine treatment to lower oxygen consumption in 3T3-L1 adipocytes or L6 myotubes, concomitant with increased AMP/ATP ratio and an increase in glycolysis. The mechanism by which berberine depleted ATP, especially in galactose medium, needs further investigation.

Our study has some limitations. Although in vitro models are good models for exploring the molecular mechanisms, they are simplistic models and will not show all the features of NAFLD and NASH in humans. NAFLD and NASH are complex conditions in which many factors contribute to the disease development. For example, the oxidative and inflammatory state produced by infiltrating macrophages (Kupffer cells) in the liver of patients with NAFLD/NASH is missing in this model. Also, hepatic stellate cells are involved in worsening the condition by producing collagen and causing fibrosis which is also missing in an in vitro model of steatosis.

## 5. Conclusions

In conclusion, despite the fact that different polyphenols differed in the strength with which they affected different mechanistic pathways, they all protected strongly against intracellular steatosis and several (resveratrol, quercetin, catechin, and cyanidin, however not kuromanin and berberine) protected against mitochondrial dysfunction and impaired aerobic energy metabolism. At least by our findings, it appears that kuromanin and berberine inhibit steatosis more specifically by modulating lipid metabolism (lipogenesis and lipid oxidation) and differ from other polyphenols in that they may not improve mitochondrial function and bioenergetics. Our study provides rationale for future studies on relative protective effects of polyphenols against NAFLD that could facilitate the path to personalized nutrition.

## Supplementary Materials

The following are available online at https://www.mdpi.com/2072-6643/11/3/541/s1, Table S1: The sequences of primers used to measure the expression of genes of interest.

## Abbreviations

**CPT1A1**	Carnitine palmitoyltransferase-1 (liver)
**FAS**	Fatty acid synthase
**GAPDH**	Glyceraldehyde-3-phosphate dehydrogenase
**MMP**	Mitochondrial Membrane Potential
**NAFLD**	Non-alcoholic fatty liver disease
**NASH**	Non-alcoholic steatohepatitis
**OXPHOS**	Oxidative phosphorylation
**p-AMPK**	Phospho-AMP-activated protein kinase
**PGC1α**	Peroxisome proliferator-activated receptor gamma coactivator 1
**PPARα**	Peroxisome proliferator-activated receptor alpha
**PPARγ**	Peroxisome proliferator-activated receptor gamma
**SIRT1**	Sirtuin 1
