# Supplementary material for: Dietary Polyphenols Protect Against Oleic Acid-Induced Steatosis in an in Vitro Model of NAFLD by Modulating Lipid Metabolism and Improving Mitochondrial Function
Source: Nutrients. 2019 Mar 3;11(3):541. doi: 10.3390/nu11030541 (PMC6471211; doi:10.3390/nu11030541)
Supplement: Supplementary file 1 [file nutrients-11-00541-s001.pdf]

Supplemental Table S1: The sequences of primers used to measure the expression of genes of interest.

**Gene**	**Species**	**Primer sequence (5'-3')**	**GenBank accession number**	**Cycles (Ct values)**
SIRT1	Human	Forward: AGT GGC AAA GGA GCA GAT TAG<br>Reverse: CTG CCA CAA GAA CTA GAG GAT AAG	NM_001142498	24
SIRT3	Human	Forward: GAA AGC CTA GTG GAG CTT CTG<br>Reverse: GGG CAG CCATCA TCC TAT TT	NM_012239	24
PPARα	Human	Forward: TCC TCG GTG ACT TAT CCT GT<br>Reverse: GCGTGG ACT CCGTAATGA TAG	NM_001001928	27
PPARγ	Human	Forward: GCCTGC ATC TCC ACCTTATTA<br>Reverse: ATC TCC ACA GAC ACG ACATTC	NM_005037	23
CPT1A1	Human	Forward: AGC GTT CTT CGT GAC GTT AG<br>Reverse: CGG CCGTGT AGT AGA GAT TTG	NM_001876	24
FAS	Human	Forward: TGT CCT GGG AGG AGT GTA AA<br>Reverse: CTG CTC CAC GAA CTC AAA CA	NM_004104	21
GAPDH	Human	Forward: ATG GGT GTG AAC CAT GAG AAG<br>Reverse: GAGTCC TTC CAC GAT ACC AAA G	NM_002046	17
